# A Stable Homoleptic Organometallic Iron(IV) Complex

**DOI:** 10.1002/chem.202002158

**Published:** 2020-09-11

**Authors:** Om Prakash, Pavel Chábera, Nils W. Rosemann, Ping Huang, Lennart Häggström, Tore Ericsson, Daniel Strand, Petter Persson, Jesper Bendix, Reiner Lomoth, Kenneth Wärnmark

**Affiliations:** ^1^ Centre for Analysis and Synthesis Department of Chemistry Lund University Box 124 Lund 22100 Sweden; ^2^ Division of Chemical Physics Department of Chemistry Lund University Box 124 Lund 22100 Sweden; ^3^ Department of Chemistry Ångström Laboratory Uppsala University Box 523 Uppsala 75120 Sweden; ^4^ Department of Physics Ångström Laboratory Uppsala University Box 528 Uppsala 751 21 Sweden; ^5^ Theoretical Chemistry Division Department of Chemistry Lund University Box 124 Lund 22100 Sweden; ^6^ Department of Chemistry University of Copenhagen Universitetsparken 5 2100 Copenhagen Denmark

**Keywords:** high-valent iron, N-heterocyclic carbenes, organometallic complexes, photophysics, transient absorption spectroscopy

## Abstract

A homoleptic organometallic Fe^IV^ complex that is stable in both solution and in the solid state at ambient conditions has been synthesized and isolated as [Fe(phtmeimb)_2_](PF_6_)_2_ (phtmeimb=[phenyl(tris(3‐methylimidazolin‐2‐ylidene))borate]^−^). This Fe^IV^ N‐heterocyclic carbene (NHC) complex was characterized by ^1^H NMR, HR‐MS, elemental analysis, scXRD analysis, electrochemistry, Mößbauer spectroscopy, and magnetic susceptibility. The two latter techniques unequivocally demonstrate that [Fe(phtmeimb)_2_](PF_6_)_2_ is a triplet Fe^IV^ low‐spin *S=*1 complex in the ground state, in agreement with quantum chemical calculations. The electronic absorption spectrum of [Fe(phtmeimb)_2_](PF_6_)_2_ in acetonitrile shows an intense absorption band in the red and near IR, due to LMCT (ligand‐to‐metal charge transfer) excitation. For the first time the excited state dynamics of a Fe^IV^ complex was studied and revealed a ≈0.8 ps lifetime of the ^3^LMCT excited state of [Fe(phtmeimb)_2_](PF_6_)_2_ in acetonitrile.

## Graphical abstract

**A stable homoleptic** organometallic Fe^IV^ complex, [Fe(phtmeimb)_2_](PF_6_)_2_ was synthesized and isolated. It is stable in the solid state and in acetonitrile at ambient temperature. The excited state of the ^3^GS [Fe(phtmeimb)_2_](PF_6_)_2_ was shown to be of ^3^LMCT nature, and shows a 0.8 ps lifetime as determined by transient absorption spectroscopy.

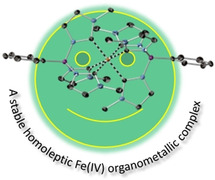

High‐valent iron complexes, both heme‐ and non‐heme, function as active key intermediates in various biological catalytic cycles and important organic transformations.^1^ This has inspired the development of synthetic chemistry of Fe^IV^ complexes.^1^, ^2^ Several heteroleptic high‐valent Fe^IV^ complexes have been reported, where the high oxidation state is stabilized by terminal π‐donating auxiliary ligands (PDALs) such as oxide, nitride, imide, isocyanide, and ketimide, with various stabilities.^3^, ^4^, ^5^, ^6^, ^7^ In particular, Fe^IV^‐oxo complexes have been found capable of various oxidative transformations.^8^ Examples of Fe^IV^ coordination compounds without stabilizing PDALs are scarce however. Besides FeF_4_, that could be isolated in a matrix,^9^ there are only few examples of more traditional Werner complexes using for example, electron‐donating dithiocarbamate ligands to stabilize the iron center in oxidation state Fe^IV^,^10^, ^11^, ^12^ or Fe^IV^ complexes containing multidentate macrocyclic tetraamide ligands,^13^ a Fe^IV^ cyclam–azide complex^14^ and a Fe^IV^ cyano‐complex based on a tridentate imino‐ligand,^15^ the two latter electrochemically generated and studied in situ. To date, the exceptional example of an entirely stable Fe^IV^ complex belongs to the class of coordination cage compounds; Fe^IV^ hexahydrazide clathrochelate.^16^ This compound shows infinite stability in both aqueous and non‐aqueous solutions as well as in the solid state, unique to synthetic Fe^IV^ complexes.

For organometallic iron complexes the electron‐donating effect of carbanions can be exploited to stabilize higher metal oxidation states which has led to a current interest in homoleptic Fe^IV^ organometallic complexes (Figure 1).^17^ Three of the reported complexes constitute Fe^IV^ tetra alkyl species (Figure 1, **1**–**3**, Table S4).18a, 18b, 18c The Fe^IV^ state of these compounds is however only accessible via disproportionation reactions and suffers from thermal instability and air sensitivity at ambient conditions (Figure 1, Table S4).18a, 18b, 18c Similarly, also the recently described organometallic Fe^IV^ complex [Fe(Cp*)_2_]^2+^ (Cp*=pentamethylcyclopentadienyl) (Figure 1, **4**) is thermally stable at room temperature only in the solid state under inert atmosphere.18d We have recently reported that the strongly σ‐donating N‐heterocyclic carbene (NHC) ligands form homoleptic Fe^II^ and Fe^III^ complexes with a *hexa*‐NHC coordination sphere leading to long photo‐induced charge‐transfer states.^19^, ^20^, ^21^ The complexes being [Fe(btz)_3_](PF_6_)_2_ 19 and [Fe(btz)_3_](PF_6_)_3_ 20 (btz^22^=3,3′‐dimethyl‐1,1′‐bis(*p*‐tolyl)‐4,4′‐bis(1,2,3‐triazol‐5‐ylidene), and [Fe(phtmeimb)_2_](PF_6_)^21^ (phtmeimb^23^ = [phenyl(tris(3‐methylimidazolin‐2‐ylidene))borate]^−^), the latter complex building on Fehlhammer's scorpionate ligand (htmeimb=[hydrido(tris(3‐methylimidazolin‐2‐ylidene))borate]^−^) and corresponding Fe^III^ complex, [Fe(htmeimb)_2_](X) (X=BPh_4_^−^, BF_4_^−^),^24^ using the analogous phtmeimb ligand developed by Smith.^23^ Another effect of the strong σ‐donor NHC ligands above is a pronounced stabilization of higher metal oxidation states apparent from the remarkably low Fe^III/II^ reduction potentials.^20^, ^21^, 24b In case of the scorpionate NHC ligand (phtmeimb), we obtained indications for the formation of a stable Fe^IV^ complex at modestly oxidizing potentials,^21^ something also observed by Fehlhammer for the Fe^III^ complex of htmeimb.24b

**Figure 1:**
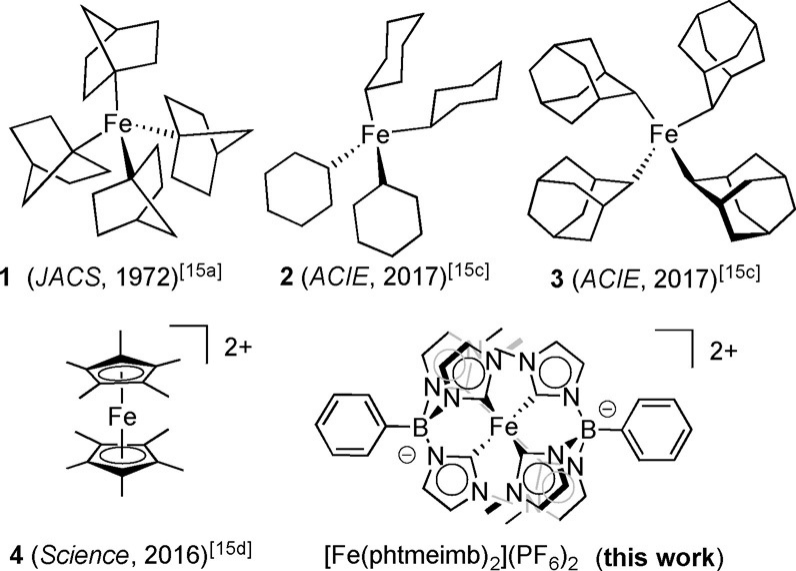
All reported organometallic homoleptic Fe^IV^ complexes.

These observations attracted our attention as examples of Fe^IV^‐NHCs, outside the electrochemical observation of the Fe^IV^ scorpionates above, are exclusively found among heteroleptic complexes, having PDALs as additionally stabilizing groups (Table S3), resulting in complexes of various stabilities.4a, 4c, 5b, 25 Here, we present the synthesis and isolation, of the stable homoleptic *hexa*‐coordinated, [Fe(phtmeimb)_2_](PF_6_)_2_ (Figure 1). The combined spectroscopic, electrochemical, magnetic and theoretical investigations characterization univocally characterize this complex as a Fe^IV^ complex. Its *S=*1 ground state undergoes a ligand‐to‐metal charge transfer (LMCT) transition and we present the dynamics of the ^3^LMCT excited state that complements our previous observations of ^3^MLCT and ^2^LMCT states with Fe^II^ and Fe^III^ NHC complexes, respectively.^19^, ^20^, ^21^

As previously reported, the Fe^III^ precursor [Fe(phtmeimb)_2_]PF_6_ undergoes a reversible one‐electron electrochemical oxidation (*E*_1/2_=0.25 V vs. ferrocene) that was assigned to the Fe^IV/III^ redox couple based on the spectroelectrochemical changes.^21^ Chemical oxidation of the red solution of [Fe(phtmeimb)_2_]PF_6_ in acetonitrile with thianthrenylhexafluorophosphate radical (*E*_1/2_=0.85 V vs. ferrocene)^26^ produced a dark green colored solution of the Fe^IV^ compound [Fe(phtmeimb)_2_](PF_6_)_2_, which could be isolated pure in 95 % yield as green crystals (see Scheme 1), as proven by the combination of ^1^H NMR spectroscopy, HR‐MS, IR, elemental analysis, scXRD analysis, electrochemistry, ^57^Fe Mößbauer spectroscopy, magnetic susceptibility and quantum chemical calculations. [Fe(phtmeimb)_2_](PF_6_)_2_ is stable in the solid state exposed to air as well as in acetonitrile solution for days at ambient temperature. In addition, the crystals of [Fe(phtmeimb)_2_](PF_6_)_2_, are stable when washed with water, however slow decomposition was observed when water was added to a solution of the complex in acetonitrile.

**Scheme 1:**
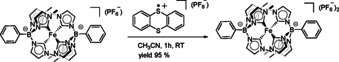
Synthesis of complex [Fe(phtmeimb)_2_](PF_6_)_2_ from [Fe(phtmeimb)_2_](PF_6_)_._

The ^1^H spectrum of [Fe(phtmeimb)_2_](PF_6_)_2_ shows highly deshielded resonances in comparison to its Fe^III^ congener and the resonances are rather well‐resolved (See Supporting Information).^21^ Dark‐green single crystals of [Fe(phtmeimb)_2_](PF_6_)_2_ suitable for scXRD analysis were grown in a saturated anhydrous acetonitrile solution of [Fe(phtmeimb)_2_](PF_6_)_2_ by slow diffusion of anhydrous diethyl ether at room temperature exposed to air (see section S4, Supporting Information, for details). The molecular structure shows an octahedral iron center surrounded by two *fac*‐tridentate phtmeimb ligands (Figure 2). The Fe−C bond lengths are 1.99 to 2.01 Å, which are close to that of the Fe^III^ congener (1.96 to 2.01 Å).^21^ Similarly, the C–Fe–C bite angles (87.3° to 87.7°) for [Fe(phtmeimb)_2_](PF_6_)_2_ are similar to the Fe^III^ congener (86.2° to 87.6°).^21^ Thus, [Fe(phtmeimb)_2_](PF_6_)_2_ exhibits a close to perfect octahedral geometry that is virtually identical to its Fe^III^ salt (details in section S4, Supporting Information). This indicates that very little structural re‐organization energy is needed when altering between the Fe^IV^/Fe^III^ oxidation states in [Fe(phtmeimb)_2_], thus facilitating rapid electron transfer processes involving this couple. The very similar Fe−C distances between the Fe^IV^ and Fe^III^ oxidation states, as well as the Fe−C distances themselves, have also been observed for a heteroleptic macrocyclic *tetra*‐NHC complex in the two oxidation states, as Fe^IV^=O and Fe^III^–O–Fe^III^ complexes.25b We suggest that the very minor difference in Fe−C bond lengths is due to the rigidity of the tridentate phtmeimb ligand and/or the covalent nature of this bond, where the influence of the formal charge differences on iron is of little importance for this bond length in the latter case.

**Figure 2:**
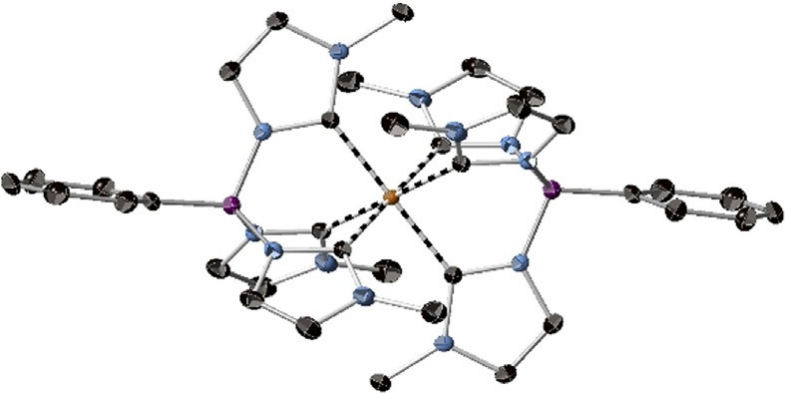
scXRD Molecular structure of the cation in [Fe(phtmeimb)_2_](PF_6_)_2_. Thermal ellipsoids are shown at 50 % probability. Hydrogen atoms, counter ions and solvent molecules are omitted for clarity. Orange=Fe; purple=B; blue=N, and grey=C.

The ^57^Fe Mößbauer spectrum of [Fe(phtmeimb)_2_](PF_6_)_2_ is shown in Figure 3 a). The isomer shift *δ* and electric quadrupole splitting Δ, of the doublet at 80 K are −0.23(1) and 3.04(1) mm s^−1^, respectively, the former in the same range as for complexes **1**–**3**, and the latter in the same range as for complex **4**,^18^ and differ from the doublet at 87 K of the Fe^III^ congener, [Fe(phtmeimb)_2_](PF_6_), −0.09 and 1.54 mm s^−1^, respectively.^21^ The combination of an unusual large Δ‐value and a negative δ‐value for the doublet supports, that this pattern emanates from Fe^IV^ triplet low spin *S=*1 in a quasi‐octahedral coordination (Section S5, Supporting Information).^27^, ^28^ The magnetic susceptibility and magnetization data for [Fe(phtmeimb)_2_](PF_6_)_2_ are reported in Figure 3 b. The distinct nesting of the magnetization curves (Figure 3 b, insert) differs from the response of the Fe^III^ precursor and clearly demonstrates the system to have an effective *S*>1/2 with a significant zero field splitting. The formulation of the complex as a low‐spin Fe^IV^ is corroborated by these magnetic data (Section S6, Supporting Information). From the magnetization data, a sizeable ZFS of *D*≈22 cm^−1^ was deduced. EPR of [Fe(phtmeimb)_2_](PF_6_)_2_, generated electrochemically from the Fe^III^ congener, does not show any EPR signal at X‐band frequencies, in either perpendicular or parallel mode (Section S7, Supporting Information). This is explained by the magnitude and likely positive sign of the determined *d*‐value which indeed precludes detection of EPR signals at any accessible frequency.

**Figure 3:**
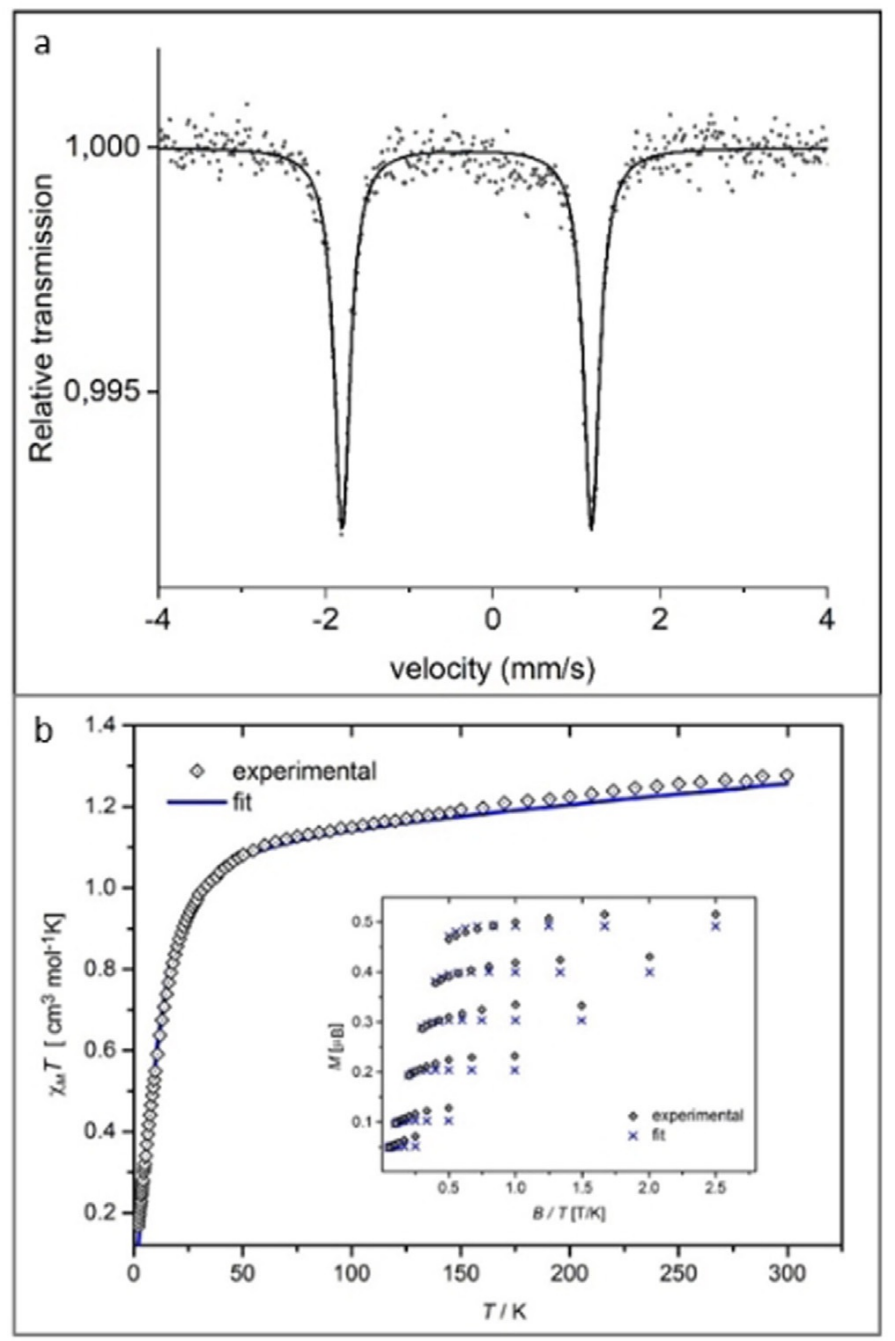
(a) ^57^Fe Mößbauer spectrum of [Fe(phtmeimb)_2_](PF_6_)_2_ at 80 K. (b) Experimental magnetic data and model fits for [Fe(phtmeimb)_2_](PF_6_)_2_. Main panel: magnetic susceptibility, represented by the *χT* product in the temperature range 2–300 K. Insert: magnetization data for the same sample recorded at *T=*2–10 K and *B*=0.5, 1.0, 2.0, 3.0, 4.0, and 5.0 T.

The electronic absorption spectrum of [Fe(phtmeimb)_2_](PF_6_)_2_ in deaerated acetonitrile (Figure 4) is dominated by a broad, intense absorption band peaking at 715 nm (*ϵ*=6850 m ^−1^ cm^−1^) with a shoulder around 810 nm, in excellent agreement with the reported spectrum obtained upon electrochemical one‐electron oxidation of the Fe^III^ precursor [Fe(phtmeimb)_2_](PF_6_).^21^ Based on electrochemical potentials of the Fe^IV/III^ couple and ligand oxidation, the low energy absorption band of the oxidized complex was previously attributed to a LMCT transition.^21^ This assignment can now be supported form the experimental data of the isolated complex and computational data (vide infra) that the transition occurs from the triplet ground state (^3^GS) with a (t_2g_^4^) electronic configuration to a ^3^LMCT state (t_2g_^5^π_L_^1^).

**Figure 4:**
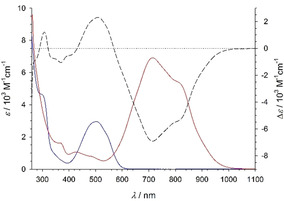
Electronic absorption spectra of [Fe(phtmeimb)_2_](PF_6_)_2_ (−) and [Fe(phtmeimb)_2_](PF_6_) (−) in deaerated acetonitrile, and the Fe^III^–Fe^IV^ differential spectrum (– – –).

In previous studies of complexes [Fe(btz)_3_]^3+^ and [Fe(btz)_3_]^2+^ we showed that the same NHC ligand set furnishes both the Fe^III^ and Fe^II^ oxidation states with exceptional lifetimes (hundreds of picoseconds) of their ^2^LMCT and ^3^MLCT excited states, respectively.^19^, ^21^ With the relative photostability of [Fe(phtmeimb)_2_](PF_6_)_2_ in acetonitrile solution (Section S8, Supporting Information), we have the opportunity to compare the excited state dynamics following LMCT excitation of a Fe^IV^ complex to the recently reported record 2.0 ns lifetime of the ^2^LMCT state of a Fe complex, featured by the Fe^III^ congener. Transient absorption spectra following 800 nm excitation of [Fe(phtmeimb)_2_](PF_6_)_2_ are shown in Figure 5. The pronounced ground state bleach (GSB), peaking at 715 nm, and the excited state absorption (ESA) at wavelengths below around 600 nm are readily rationalized in terms of the spectral differences arising from the Fe^IV^ to Fe^III^ reduction. The additional excited state absorption at wavelengths above around 900 nm can be attributed to the oxidation of the phtmeimb ligand in analogy to the spectrum of the LMCT excited state of [Fe(phtmeimb)_2_](PF_6_) that involves the same ligand oxidation.^21^ The transient‐absorption spectrum hence corroborates the assignment of the 715 nm absorption band of [Fe(phtmeimb)_2_](PF_6_)_2_ to an LMCT transition from the t_2g_^4^ low‐spin ground state (^3^GS) to a t_2g_^5^ π_L_^1^ (^3^LMCT) excited state. Most of the transient absorption decays with a ≈0.8 ps lifetime, indicating fast deactivation of the ^3^LMCT state accompanied by similarly fast ground state recovery. Only a minor component with a 16 ps lifetime that contributes to the ground state recovery and the transient absorption between 850 and 1000 nm points to the involvement of additional states in the deactivation of the ^3^LMCT excited state.

**Figure 5:**
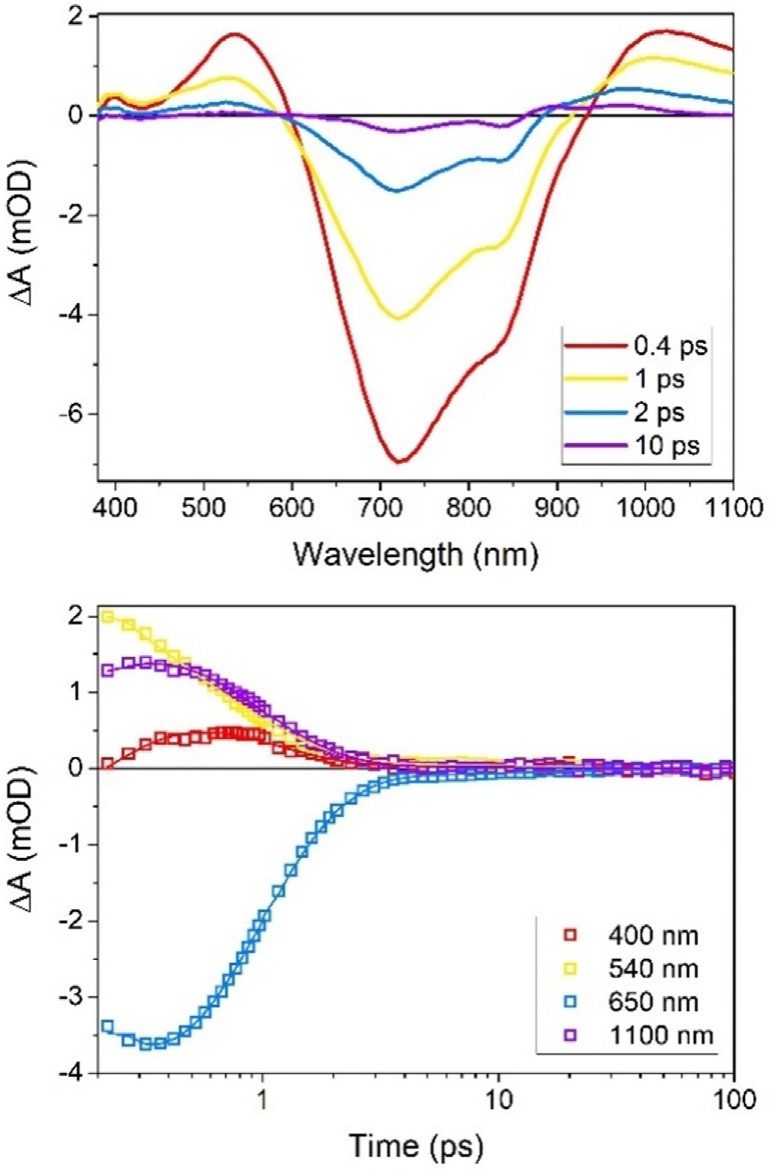
Transient absorption results for [Fe(phtmeimb)_2_](PF_6_)_2_ in deaerated acetonitrile—full spectral decays (top), and kinetics at 400 nm, 540 nm, 650 nm, and 1100 nm (bottom).

Quantum chemical calculations of different relaxed spin states corroborate the nature and structure of the ground state as a triplet state. Figure 6 shows the calculated ground state spin density of the ^3^[Fe(phtmeimb)_2_]^2+^ complex, which, together with the calculated Mulliken spin density on the iron for this state (Table 1) supports an assignment of the ground state as a triplet Fe^IV^ complex with (t_2g_)^4^ character, with some admixing of the frontier molecular orbitals with NHC‐π contributions.

**Figure 6:**
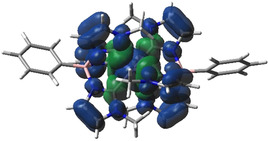
Calculated spin density for the optimized lowest energy state of ^3^[Fe(phtmeimb)_2_]^2+^. Spin‐up density is in blue and spin down in green.

**Table 1:** Quantum chemically calculated properties of [Fe(phtmeimb)_2_]^2+^ for fully optimized states of different overall spin multiplicities. Calculated properties include relative total energies (*E*_*r*el_), average Fe−C bond lengths (*R*_av_(Fe‐C)), and Mulliken Spin densities on the central metal ion (Fe Spin)

State	*E*_rel_ [eV]	*R*_av_(Fe−C) [Å]	Fe Spin
^1^[Fe(phtmeimb)_2_]^2+^	1.26	2.000	0.00
^3^[Fe(phtmeimb)_2_]^2+^	0.00	2.021	2.00
^5^[Fe(phtmeimb)_2_]^2+^	1.84	2.129	3.41

The synthesized and isolated [Fe(phtmeimb)_2_](PF_6_)_2_ complex, is stable in the solid state and acetonitrile solution at ambient conditions. The facile tunability of the NHC ligand systems provide potential for stabilization of high‐valent metal complexes in general. We have built on Fehlhammer's initial observation,^24^ and developed an organometallic high‐valent Fe^IV^ NHC complex without additional stabilizing π‐donating ligands,4a, 4c, 5b, 25a, 25b, 25d stable at ambient conditions, utilizing the strongly σ‐donating mono‐anionic facial *tris*‐NHC scorpionate ligand phtmeimb developed by Smith.^23^ The excited state dynamics of homoleptic [Fe(phtmeimb)_2_](PF_6_)_2_ was studied, constituting the first of its kind study of a Fe^IV^ complex. We observed the fundamental difference in excited state lifetimes for Fe^III^‐^2^LMCT (2 ns, 2.1 eV) vs. Fe^IV^‐^3^LMCT (0.8 ps, <1.5 eV) states, which deserves further investigation beyond the scope of the present paper. Moreover, while advanced designs and studies are needed for a better understanding of Fe^IV^ NHC complexes, modifications that aim at further prolonging the ^3^LMCT state through subtle tuning of the energy levels are also in progress. Finally, having access to stable Fe^IV^ species, with long lived ^3^LMCT state that can take part in redox/photocatalytic processes/cycles, is an appealing area for future exploration.

## Conflict of interest

The authors declare no conflict of interest.

## Supporting information

As a service to our authors and readers, this journal provides supporting information supplied by the authors. Such materials are peer reviewed and may be re‐organized for online delivery, but are not copy‐edited or typeset. Technical support issues arising from supporting information (other than missing files) should be addressed to the authors.

Supplementary
