# Supplementary material for: A Stable Homoleptic Organometallic Iron(IV) Complex
Source: Chemistry. 2020 Sep 11;26(56):12728–32. doi: 10.1002/chem.202002158 (PMC7590184; doi:10.1002/chem.202002158)
Supplement: Supplementary file 1 — Supplementary [file CHEM-26-12728-s001.pdf]

Chemistry–A European Journal

Supporting Information

A Stable Homoleptic Organometallic Iron(IV) Complex

Om Prakash,[a] Pavel Chábera,[b] Nils W. Rosemann,[b] Ping Huang,[c] Lennart Häggström,[d] Tore Ericsson,[d] Daniel Strand,[a] Petter Persson,*[e] Jesper Bendix,*[f] Reiner Lomoth,*[c] and Kenneth Wärnmark*[a]

Contents:

1. **S1 Experimental Section**…………………

    1.1 S1.1 General experimental details

    1.2 S1.2 Synthesis of $[Fe^{IV}(phtmeimb)_2](PF_6)_2$

2. **S2 ^{1}H NMR Spectrum**…………………
3. **S3 HR-MS Spectrum**…………………
4. **S4 Single Crystal X-ray diffraction**…………………
5. **S5 Mößbauer spectroscopy**…………………
6. **S6 Magnetic susceptibility and magnetization measurements**…………………
7. **S7 Electron paramagnetic resonance measurements**…………………
8. **S8 Photostability**…………………
9. **S9 Transient absorption spectroscopy**…………………
10. **S10 Quantum Chemistry**…………………
11. **S11 Comparison tables of reported Organometallic Fe(IV) complexes……….**
12. **S12 References**…………………

S1 Experimental Section

S1.1 General experimental details

^{1}H and ^{13}C NMR spectra were recorded on a Bruker Avance II 400 MHz NMR spectrometer. Chemical shifts (δ) are reported to the shift-scale calibrated with the residual NMR solvent; CD_3CN (1.94 ppm for ^{1}H NMR spectra). Electrospray ionization–high resolution mass spectrometry (ESI–HRMS) and atmospheric pressure chemical ionization (APCI) for mass spectrometry were recorded on a Waters Micromass Q-Tof micro mass spectrometer. Infrared spectra were recorded as the neat compound on a Bruker, Alpha-P FTIR spectrometer. Melting points of the compounds were measured on a Stuart Scientific Melting Point Apparatus-SMP3. Elemental analyses were performed by Mikroanalytisches Laboratorium KOLBE (Mülheim an der Ruhr, Germany).

Thianthrenylhexafluorophosphate and bis(Tris(3-methylimidazol-1-ylidene)(phenyl)borate)$Fe^{(III)}$ hexafluorophosphate was synthesized using published methods.[S1-2] Anhydrous CH_3CN and diethyl ether were obtained from a PureSolv PSM-768 and Braun SPS-800 system, respectively.

S1.2 Synthesis of $[Fe^{IV}(phtmeimb)_2](PF_6)_2]$

Bis(tris(3-methylimidazol-1-ylidene)(phenyl)borate)$Fe^{(III)}$hexafluorophosphate (0.086 g, 0.10 mmol) and thianthrenylhexafluorophosphate (0.0380 g, 0.105 mmol) were dissolved in dry acetonitrile (10 mL) at room temperature. The reaction mixture was stirred for 1h at room temperature. The so-formed green solid was precipitated by the addition of dry diethyl ether (100 mL) to the dark green solution, until the supernatant solution appeared colorless. The resulting green residue was filtered using glass sinter (porosity #4) and washed with dry diethyl ether. The resulting green complex was recrystallized in dry CH_3CN via slow diffusion of dry diethyl ether in the dark under air to yield green crystals (0.096 g, 95%). ^{1}H NMR (400 MHz, CD_3CN, 10 mM): δ (ppm) 64.63 (b, 4H, $H_{Ph\text{-}c}$), 6.12-5.81 (m, 6H, $H_{Ph\text{-}a,\,b}$), 2.87 (s, 18H, $H_{Me\text{-}f}$) -60.84 (b, 6H, $H_{Im\text{-}d}$) -71.21 (b, 6H, $H_{Im\text{-}e}$) ;^{13}C NMR (100.3 MHz, CD_3CN, 30mM): δ (ppm); 135.1, 128.7, 128.1, 126.0, 124.6, 123.6, 14.7; IR (thin layer film) ν (cm^{-1}) 1658, 1540, 1450, 1402, 1335, 1294, 1200, 1177, 1123, 1045, 1025, 882, 831, 794, 766, 733, 707, 555; MP: 349; ESI–HRMS (m/z): $[(C_{36}H_{40}N_{12}B_2Fe+PF_6)]^+$ calcd for $C_{36}H_{40}N_{12}B_2FePF_6$, 863.2676; found, 863.2679; Elemental

analysis: (% calcd, % found for $C_{36}H_{40}N_{12}P_2B_2F_{12}Fe$): C (42.89, 42.92), H (4.00, 4.07), N (16.67, 16.65).

S2 ^1H NMR Spectrum

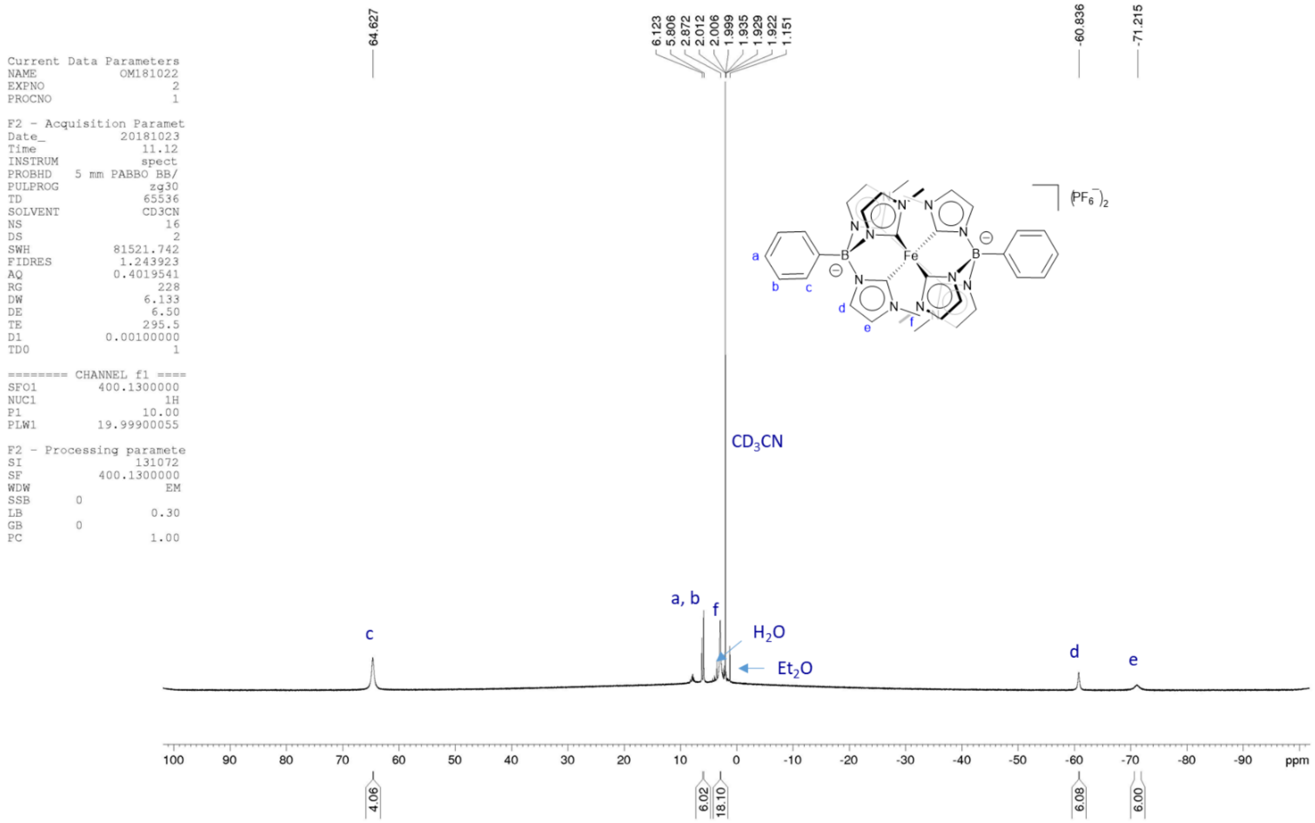

Figure S1. ^1H NMR (400 MHz) spectrum of $[Fe(phtmeimb)_2](PF_6)_2$ (12 mM) in CD_3CN.

S3 HR-MS Spectrum

Elemental Composition Report

Page 1

Single Mass Analysis
Tolerance = 2.0 mDa / DBE: min = -1.0, max = 100.0
Element prediction: Off
Number of isotope peaks used for i-FIT = 3

Monoisotopic Mass, Even Electron Ions
1068 formula(e) evaluated with 3 results within limits (all results (up to 1000) for each mass)
Elements Used:
C: 0-50 H: 0-100 N: 12-15 O: 0-2 F: 5-6 P: 1-1 56Fe: 0-1 B: 0-2

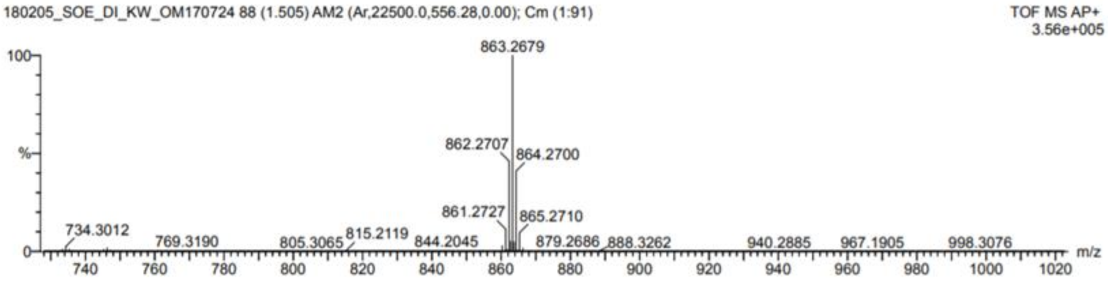

Mass	Calc. Mass	mDa	PPM	DBE	Conf(%)	Formula
Minimum:				-1.0		
Maximum:		2.0	10.0	100.0		
863.2679	863.2679	0.0	0.0	29.5	0.00	C41 H34 N12 O2 F5 P B
	863.2676	0.3	0.3	21.5	0.00	C36 H40 N12 F6 P 56Fe B2
	863.2671	0.8	0.9	30.5	100.00	C43 H34 N12 F6 P

Figure S2. ESI HR-MS spectrum of $[Fe(phtmeimb)_2](PF_6)_2$.

Elemental Composition Report

Page 1

Single Mass Analysis
Tolerance = 2.0 mDa / DBE: min = -1.0, max = 100.0
Element prediction: Off
Number of isotope peaks used for i-FIT = 3

Monoisotopic Mass, Even Electron Ions
1068 formula(e) evaluated with 3 results within limits (all results (up to 1000) for each mass)
Elements Used:
C: 0-50 H: 0-100 N: 12-15 O: 0-2 F: 5-6 P: 1-1 56Fe: 0-1 B: 0-2

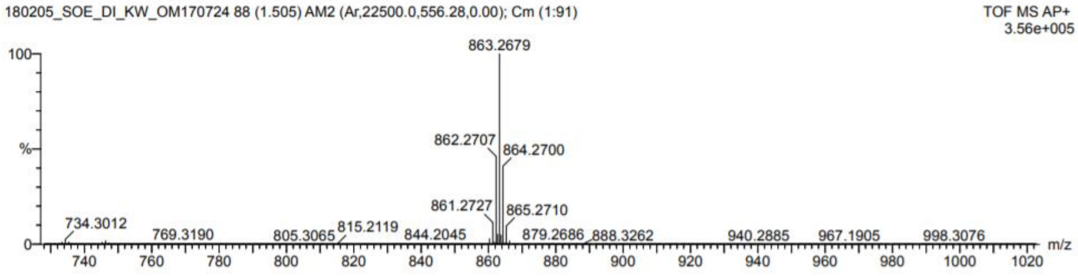

Mass	Calc. Mass	mDa	PPM	DBE	Conf(%)	Formula
Minimum:				-1.0		
Maximum:		2.0	10.0	100.0		
863.2679	863.2679	0.0	0.0	29.5	0.00	C41 H34 N12 O2 F5 P B
	863.2676	0.3	0.3	21.5	0.00	C36 H40 N12 F6 P 56Fe B2
	863.2671	0.8	0.9	30.5	100.00	C43 H34 N12 F6 P

Figure S3. APCI HR-MS spectrum of $[Fe(phtmeimb)_2](PF_6)_2$.

S4 Single Crystal X-ray diffraction

A single crystal, grown by slow diffusion of dry diethyl ether into a solution of $[Fe(phtmeimb)_2](PF_6)_2$ in dry acetonitrile in air, covered in paratone oil was mounted on a glass fiber and rapidly transferred to the nitrogen cold stream of the diffractometer. Data collection was performed at 110 K on an Agilent Xcalibur Sapphire3 diffractometer equipped with a Mo *Kα* high-brilliance IμS radiation source (λ = 0.71073 Å) and an Oxford Cryosystems low temperature device. The structure was solved and refined using SHELX 2016/4.[S3] Non-hydrogen atoms were refined anisotropically.

Table S1. Crystal data and structure refinement for $[Fe(phtmeimb)_2](PF_6)_2]^{\cdot}(MeCN)_2$.

Chemical formula	$C_{36}H_{40}B_2FeN_{12}$, $2(F_6P)$, $2(C_2H_3N)$	
Formula mass	4361.26	
Crystal size	0.2 x 0.2 x 0.2 mm^3	
Crystal habit	Dark green, irregular	
Temperature	110(2) K	
Wavelength	0.71073 Å	
Crystal system	Orthorhombic	
Space group	P c c n	
Unit cell dimensions	*a* = 21.5565(5) Å	α = 90°.
	b = 11.8361(2) Å	β = 90°.
	c = 19.3448(4) Å	γ = 90°.
Cell unit volume	4935.73(17) Å^3	
Number of formula units per cell, *Z*	1	
Density (calculated)	1.467 Mg/m^3	
Absorption coefficient	0.462 mm^{-1}	
F(000)	2232	
Reflections collected	39649	
Independent reflections	6170 [$R_{(int)}$ = 0.0517]	
Data / restraints / parameters	6170 / 0 / 325	
Goodness-of-fit on F^2	1.043	
Final R indices [$I>2\sigma(I)$]	R_1 = 0.0468, $wR2$ = 0.1188	
R indices (all data)	R_1 = 0.0571, $wR2$ = 0.1261	
Largest diff. peak and hole	0.768 and -0.625 e.Å^{-3}	
CCDC	1961137	

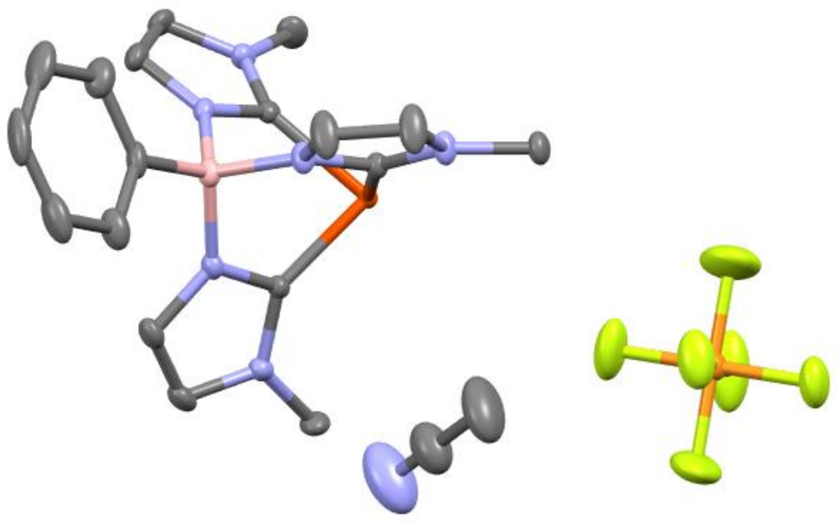

Figure S4. The asymmetric unit of [Fe(phtmeimb)$_2$](PF$_6$)$_2$]·2(MeCN). Gray = carbon atom, blue = nitrogen atom; pink = boron atom; red = iron atom; orange = phosphorous atom; green = fluorine atom. Thermal ellipsoids are shown at 50% probability. Hydrogen atoms are omitted for clarity.

Table S2. Selected bond distances and bond angles in [Fe(phtmeimb)$_2$](PF$_6$)$_2$.[a]

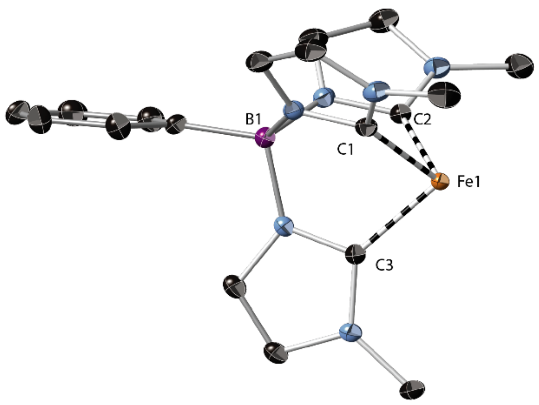

Selected angles /°		*Selected distances* /Å	
C1 – Fe1 – C2	87.25(7)	Fe1 … C1	2.0104(18)
C1 – Fe1 – C3	87.72(7)	Fe1 … C2	2.0056(18)
C2 – Fe1 – C3	87.64(7)	Fe1 … C3	1.9913(18)
Fe1 – B1 – C4	174.26(13)	Fe1 … B1	3.200(2)

[a] Distances and angles were measured using Olex2 v1.2.8.

S5 Mößbauer spectroscopy

^{57}Fe Mößbauer measurements were carried out in an Oxford Instrument flow cryostat at temperatures between 80K and 295K using a ^{57}Co<u>Rh</u> source. The powder sample was held between plastic holder to form an absorber with a concentration of 25mg/cm^2. Calibration spectra were recorded from an iron metal foil. The resulting spectra were analysed using a least squares Mößbauer fitting program.

The Mößbauer spectra of $[Fe(phtmeimb)_2](PF_6)_2$ at 80K and at 295K are very similar and are dominated by a symmetric doublet (Figure 3a). The isomer shift δ and electric quadrupole splitting Δ of the doublet at 80K are -0.23(1) mm/s and 3.04(1) mm/s (errors for all hyperfine parameters are 0.01 mm/s), respectively. The Lorentzian line width Γ for the strong intensity doublet was found to be 0.26(1) mm/s. The combination of an unusual large electric quadrupole splitting Δ and a negative isomer shift value δ for the doublet supports, that this pattern emanates from Fe(IV) *triplet* low spin S=1 in a quasi-octahedral coordination.[S4-5] From the earlier studies of similar compounds, it is natural to assume that the low intensity signal in the Mößbauer spectra emanates from low spin, S=0, Fe(II).[S2,S6] The strong temperature dependence of the doublet intensities (at 296 K the spectral intensities were found to be 99% and 4%, respectively) and reveals furthermore a difference in Debye temperatures θ_D for the two Fe valences in the sample in line with earlier findings.[S2,S6] The true Fe(IV) and Fe(II) atomic concentrations in the present sample of $[Fe(phtmeimb)_2](PF_6)_2$ can thus be approximated to 99(1)% and 1(1)%, respectively. The low intensity Fe(II) signal likely originates from an impurity different from $[Fe(phtmeimb)_2](PF_6)_2$.

S6 Magnetic susceptibility and magnetization measurements

The magnetic data were acquired on a Quantum-Design MPMS-XL SQUID magnetometer. Susceptibility data were acquired in a static field of 0.1 T. Magnetization data were obtained with selected fields from 0 to 5 T and in the temperature range 2-10 K. The polycrystalline samples were measured on a pressed pellet. The diamagnetic contribution to the sample moment from the sample holder and sample was absorbed in the total diamagnetism/TIP correction. Data were processed and modelled by use of the Mag Propprogram.[S7]

The magnetic susceptibility and magnetization data for $[Fe(phtmeimb)_2](PF_6)_2$ are reported in Figure 3b. The distinct nesting of the magnetization curves (Figure 3b, insert) differs from the response of the Fe(III) precursor and clearly demonstrates the system to have an effective *S*>½ with a significant zero field splitting. The formulation of the complex as a low-spin Fe(IV) is corroborated by these magnetic data. The temperature dependence of the χT product (Figure 3b, main panel) can be modelled in a minimalistic way using a triplet spin state with an axial *g*-tensor, split by an axial zero-field splitting (ZFS) parameter (*D*). The pertinent Hamiltonian is given in eq. S1.

$$\hat{H} = g_z^{eff} \mu_B B_z \cdot S_z + g_{x,y}^{eff} \mu_B B_{x,y} \cdot S_{x,y} + D\left(S_z^2 - \frac{2}{3}\right) + TIP \qquad \text{(eq. S1)}$$

This simplistic modelling, neglecting the only partially quenched orbital contribution, imposes a sizable TIP (TIP = Temperature-independent paramagnetism) ($4.7 \cdot 10^{-4}$ cm^3mol^{-1}) and anisotropic effective *g*-values ($g_{x,y}^{eff}=1.88$; $g_{x,y}^{eff}=2.40$) on the modelling to obtain a good fit with a ZFS of D=21.7 cm^{-1} (Figure 3b, main panel). The overall magnetic behaviour is similar to that of other approximately trigonal low-spin systems with partially quenched orbital contributions.[S8] The treatment is validated by a decent reproduction of the magnetization data using the same global parameter values (Figure 3b, insert panel).

S7 Electron paramagnetic resonance measurements

X-band EPR measurements were performed on a BrukerELEXYS E500 spectrometer equipped with a SuperX EPR049 microwave bridge and a cylindrical TE_{011} ER 4122SHQE cavity. The temperature was controlled using an Oxford Instruments ESR 900 flow cryostat. EPR spectra were analyzed using the XEPR software package. Helium temperature EPR spectra were recorded on a Bruker E580-ELEXSYS spectrometer equipped with an Oxford 900 liquid helium cryostat and an ITC 503 temperature controller. An ER4116DM dual mode X-band resonator of rectangular type (TE_{102} for perpendicular and TE_{012} for parallel mode) was used for measurements. Modulation frequency of 100 kHz was applied for all spectral recordings. 10 Samples of $[Fe(phtmeimb)_2](PF_6)_2$ (ca. 1 mM) were dissolved in argon purged, dry acetonitrile and transferred into EPR tubes under dim-light. Samples in different oxidation states were obtained by exhaustive controlled potential electrolysis at -1.48 V, -0.78 V, and +0.62 V vs. fc that yield the Fe(II), Fe(III) and Fe(IV) state, respectively. Samples of 150 µl were transferred from the electrolysis cell to argon filled EPR tubes using an argon flushed gas tight syringe. All samples 15 were stored in liquid nitrogen and in darkness before EPR examination. Neither $[Fe(phtmeimb)_2](PF_6)_2$ nor any of the electrolyzed samples showed any EPR signal in perpendicular mode, irrespective of temperature (4 to 25 K) and microwave power (e.g. 0.2 mW and 0.8 mW). Also in parallel mode at liquid helium temperature all samples were EPR silent.

S8 Photostability

The absorbance measurements were performed using a Perkin Elmer Lambda 1050 UV/Vis/NIR instrument.

To evaluate the stability of $[Fe(phtmeimb)_2](PF_6)_2$ we performed long-term steady-state absorption measurements. For comparison we performed the same measurements using the reduced form $[Fe(phtmeimb)_2](PF_6)$ and the well-established tris(bipyridine)ruthenium(II) complex ($[Ru(bpy)_3](PF_6)_2$). For each sample a solution of freshly distilled acetonitrile and the complex was prepared. The samples were stored in standard 1 mm optical path-length cuvettes that had been sealed with a stopper and additional parafilm. Concentrations of the solutions were determined based on the absorbance measurements directly after sample preparation. For the Fe(IV) complex a concentration of 160 µM was estimated based on the absorbance at 700 nm (ε_{700nm}=6900 1/Mcm). The concentration of the Fe(III) solution was estimated to be at 50µM based on the absorbance at 502 nm (ε_{502nm}=2950 1/Mcm).[S2] Concentration of the Ru(II) complex was 19 µM based on the absorption measured at 451 nm (ε_{451nm}=14600 1/Mcm).[S9] Over the term of two weeks we performed steady-state absorption measurements on regular intervals. Between the measurements the samples were illuminated using a standard (11 W) compact fluorescent lamp mounted directly above the cuvettes (See Figure S5). During the two weeks no significant change of the filling level in the cuvette was noticeable. The results of the measurements are summarized in Figure S6. For the Fe(IV) complex we observe a quenching of the absorbance around 700 nm accompanied by an increase of absorbance in the region of 500 nm. As known from spectroelectrochemistry measurements we assign this change to a reduction from the Fe(IV) to Fe(III) species.[S2] The Fe(III) sample shows a slight increase in absorbance on the low energy shoulder. For the Ru(II) sample we observe an increase on the low energy shoulder of the MLCT transition and a decrease of the latter. To underline these observations, the temporal evolution of the absorbance maximum corresponding to the main transition of all three complexes is plotted (Figure 6d).

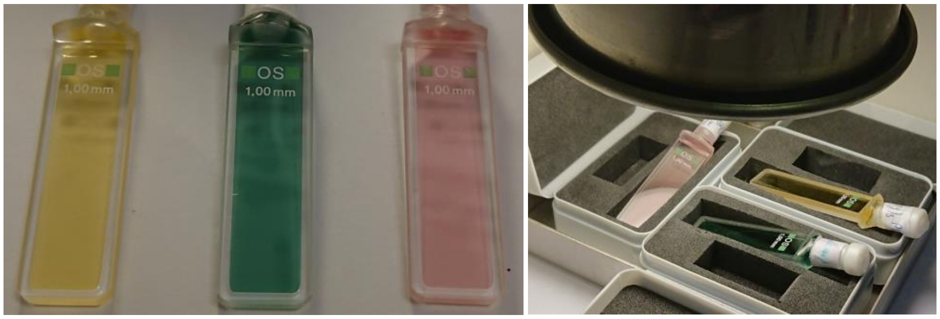

Figure S5. Photograph of [Fe(phtmeimb)$_2$](PF$_6$)$_2$, [Fe(phtmeimb)$_2$](PF$_6$) and [Ru(bpy)$_3$](PF$_6$)$_2$ under illumination. **Left)** From left to right: [Ru(bpy)$_3$](PF$_6$)$_2$ (yellow), [Fe(phtmeimb)$_2$](PF$_6$)$_2$ (green) and [Fe(phtmeimb)$_2$](PF$_6$) (light red) in acetonitrile. **Right)** Samples under compact fluorescent lamp for stability test.

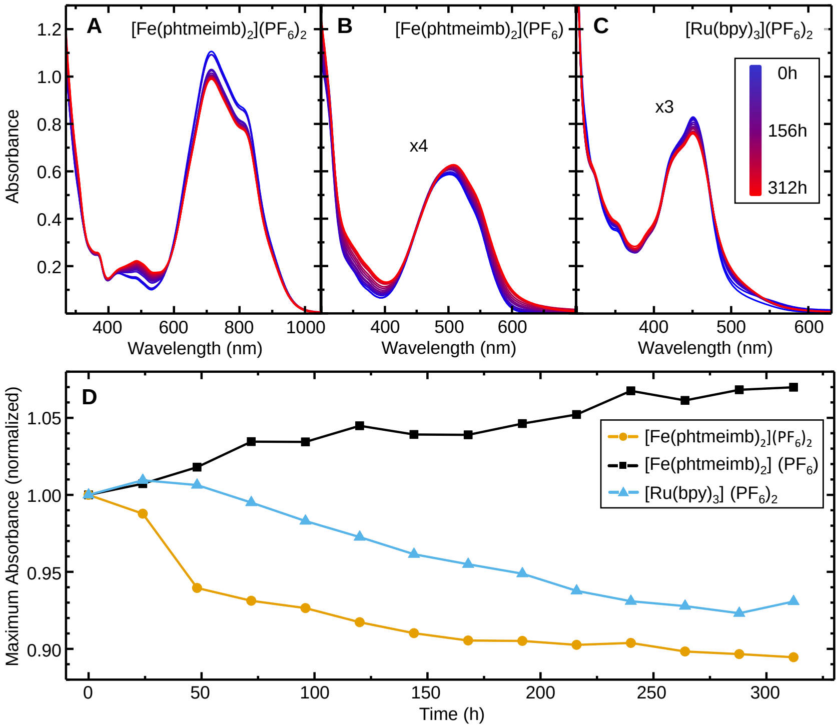

Figure S6. Long term absorbance study of [Fe(phtmeimb)$_2$](PF$_6$)$_2$, [Fe(phtmeimb)$_2$](PF$_6$) and [Ru(bpy)$_3$](PF$_6$)$_2$. (**A**) Absorbance spectra of [Fe(phtmeimb)$_2$](PF$_6$)$_2$ over the course of 312 hours. (**B**) Development of the absorbance spectra of [Fe(phtmeimb)$_2$](PF$_6$) over a period of 312 hours, scaled by a factor of 4. (**C**) Absorbance spectra of [Ru(bpy)$_3$](PF$_6$)$_2$ over a period of 312 hours, scaled by a factor of 3. The color code in all tiles ranges from the start of the measurement series (blue) to the last measurement 312 h later (red). (**D**) Changes of the maximum absorbance of [Fe(phtmeimb)$_2$](PF$_6$)$_2$ (orange circles),

$[Fe(phtmeimb)_2](PF_6)$ (black squares) and $[Ru(bpy)_3](PF_6)_2$ (light blue triangles) normalized to the value taken at zero hour. For each complex only the strongest transition is considered, i.e., ~715 nm for $[Fe(phtmeimb)_2](PF_6)_2$, ~502 nm for $[Fe(phtmeimb)_2](PF_6)$ and ~450 nm for $[Ru(bpy)_3](PF_6)_2$.

S9 Transient absorption spectroscopy

Broadband transient absorption experiments were performed using a Ti:Sapphire amplified laser system (Spitfire XP Pro, Spectra Physics) operating at a 1 kHz repetition rate, producing ~80 fs pulses centered at 796 nm. A small fraction of the fundamental beam was used to excite the sample with typical fluency not exceeding 10^{14} photons per pulse per cm^2. A white-light super-continuum was used as the probe beam, generated by focusing an IR signal from OPA (Topas-C, Light Conversion) into a 3 mm CaF_2 plate. The desired timing between excitation and probe pulses was achieved by a computer-controlled delay line (Aerotech). The pump and the probe beams were overlapped in the sample volume with their relative polarization set to the magic-angle (54.7º) by a Berek polarization compensator placed in the pump beam path. The sample of $[Fe(phtmeimb)_2](PF_6)_2$ was placed in a quartz 1 mm path length cuvette with an automated sample mover to avoid sample photodamage. The probe and reference beams were collimated on the entrance apertures of a prism-based spectrograph and collected by linear diode-array (Pascher Instruments). Global fitting of the transient absorption datasets was carried out using an in-house developed Python routine.

S10 Quantum Chemistry

Optimized geometries and electronic structure properties were calculated using the B3LYP*[S10] modified functional version of the standard B3LYP[S11] hybrid functional together with a standard 6-311G(d,p) basis set[S12] and a polarizable continuum model (PCM)[S13] description for an acetonitrile solvent. Full optimizations of different spin states were performed at the density functional theory (DFT) or unrestricted DFT (UDFT) level of theory as appropriate and without any symmetry constraints. All calculations were performed using the Gaussian09 program.[S14]

S11 Comparison tables of reported Organometallic Fe(IV) complexes

Table S3. Comparison table for organometallic Fe(IV) heteroleptic complexes.

S. No.	Organometallic Fe(IV) heteroleptic Complex	Stability in		Charact-erized by	Crystal structure / Fe-C bond length (Å)	Electronic state	Spin state of ground state	Magnetic susceptibility	Mössbauer shift (δ) and quadrupole splitting (ΔE_Q)	Redox-potential	λmax (nm) [ε($M^{-1} cm^{-1}$)]	Excited state dynamics
		Solution	Solid state									
1[S15]	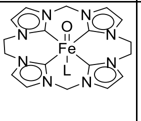 L = CH_3CN, EtCN	Stable (CH_3CN) at -40 °C for more than one month At RT few hours	ND	HR-MS, single crystal X-ray, and Mössba-uer	Determine / 1.979(5) 1.980(5) 2.037(5) 2.045(5)	t_{2g}^4	S = 1	The $\chi_M T$ value at 200 K of 0.98 cm^3 K mol^{-1} D=+16.4 cm^{-1}	-0.13 mm s^{-1} and 3.08 mm s^{-1}	ND	400 [200]	ND
2[S16]	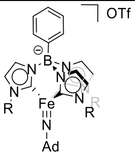 R = mesitylene	ND	air-stable can be heated for days at 100 °C	^1H NMR, HR-MS, and single crystal X-ray	Determine / 1.978(3) 1.956(9) 1.972(3)	ND	S =1	μ_{eff} = 2.7(3) BM) by Evans method	ND	$E_{1/2}$ = -0.98 V (Cp_2Fe^+/Cp_2Fe, Fe^{IV}/Fe^{III})	ND	ND
3[S17]	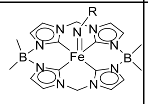 R = *t*Bu, Ad, DiPP	ND	Thermally stable at RT, but exhibit extreme water sensitivity	^1H, ^{13}C NMR, HR-MS, IR, single crystal X-ray, and Mössba-uer	Determine / 1.9331(14) 1.9865(14) 1.9293(13) 1.9802(13) 1.9868(14) 1.9730(13) 1.9963(14)	ND	Low-spin (S=0) intermediate-spin (S=1)	ND	-0.18 mm s^{-1}, -0.18 mm s^{-1}, -0.11 mm s^{-1} and 0.97 mm s^{-1}, 1.11 mm s^{-1}, 2.67 mm s^{-1}	ND	ND	ND
4[S18]	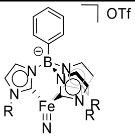 R = *t*Bu, mesitylene	ND	ND	^1H, ^{15}N NMR, HR-MS, and single crystal X-ray	Determine / 1.885(6) 1.921(6) 1.915(1) 1.928(1) 1.928(1)	ND	ND	ND (*t*Bu, Mes)	ND	$E_{1/2}$ = -1.91 V (Fc/Fc^+, Fe^{IV}/Fe^{III}) (Mes) ND(*t*Bu)	478, 324, 265 [1230], [7292], [8708] (*t*Bu) ND (Mes)	ND
5[S19]	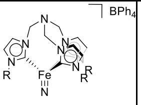 R = mesitylene, xylene	air and moisture stable (THF) at RT	air and moisture stable at RT	^1H, ^{13}C NMR, CHN analysis, single crystal X-ray, and Mössba-uer	Determine / 1.987(3) 1.920(7) 1.966(4) 1.955(5) 1.952(4) 1.946(4) 2.006(8)	$(d_{xy})^2$ $(d_{x2-y2})^2$ $(d_{z2})^0(d_{xz})^0(d_{yz})^0$	S =0	ND	-0.27 mm s^{-1} and 6.04 mm s^{-1} (Mesitylene)	ND	520 [1980] (Mes)	ND
6[S20]	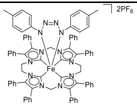	air stable (CH_3CN) at RT	air stable at RT	^1H, ^{13}C NMR, HR-MS, IR, CHN analysis, single crystal X-ray, and Mössba-uer	Determine / 1.952(3) 1.987(3) 1.954(3) 2.011(2)	ND	a low spin (S = 0)	ND	-0.01 mm s^{-1}, and 0.62 mm s^{-1}	$E_{1/2}$ = -1.05 V (Fc/Fc^+, Fe^{IV}/FeIII)	420 [9300]	ND
7[S21]	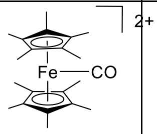	Not stable in organic solvents	Stable at RT under inert atmosphere	^1H, ^{13}C NMR, single crystal X-ray, and Mössba-uer	Determine / 2.13-2.22	$(dz_2)^2$ $(dxz)^2$	S = 0	ND	0.42 mm s^{-1} and 3.22 mm s^{-1}	ND	ND	ND

ND = No data available; [ε] = Extinction coefficient

Table S4. Comparison table for organometallic Fe(IV) homoleptic complexes.

S. No.	Organometallic Fe(IV) homoleptic Complex	Stability in		Characterized by	Crystal structure/ Fe-C bond length	Electronic state	Spin state of ground state	Magnetic susceptibility	Mössbauer shift (δ) and quadrupole splitting (ΔE_Q)	Redox-potential	λmax (nm) [ε (M^{-1} cm^{-1})]	Excited state dynamics
		Solution	Solid state									
1[S22]	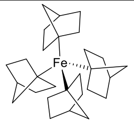	Stable (toluene solvent) at -20 °C under argon	stable at -20 °C under argon	^{1}H, ^{13}C NMR, IR, single crystal X-ray, and Mössbauer	Determine / 2.002(14) 1.984(7) 2.002(9)	ND	Low-spin S = 0	μ_{eff} = 0 BM	-0.28 mm s^{-1} and 0.15 mm s^{-1}	ND	ND	ND
2[S23]	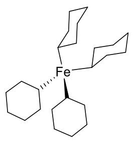	stable (toluene solvent) at -20 °C under argon	stable at -20 °C under argon	^{1}H, ^{13}C NMR, CHN analysis, single crystal X-ray, and Mössbauer	Determine / 1.931(1) 1.928(1)	ND	Low-spin S = 0	ND	-0.29 mm s^{-1} and 0.28 mm s^{-1}	ND	277 [310]	ND
3[S23]	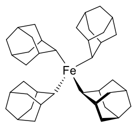	stable (toluene solvent) at -20 °C under argon	stable at -20 °C under argon	CHN analysis, and Mössbauer	ND	ND	ND	ND	-0.13 mm s^{-1} and 0.69 mm s^{-1}	ND	ND	ND
4[S24]	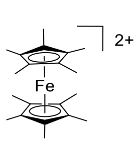	Not stable in organic solvents	Stable at RT under inert atmosphere	single crystal X-ray, and Mössbauer	Determine / 2.12-2.15	$e_{2g}^3a_{1g}^1$	S = 1	μ_{eff} = 3.04 BM	0.59 mm s-1 and 2.00 mm s^{-1}	ND	ND	ND
5 this work	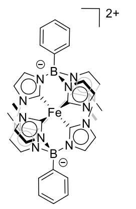	air and moisture stable (CH_3CN) at RT	air and moisture stable at RT	^{1}H, ^{13}C NMR, HR-MS, IR, CHN analysis, single crystal X-ray, and Mössbauer	Determine / 2.0104(18) 2.0056(18) 1.9913(18)	t_{2g}^4	Low-spin S = 1	$D \approx 22$ cm^{-1}	-0.23 mm s^{-1} and 3.04 mm s^{-1}	($E_{1/2}$= 0.25 vs. ferrocene) Fe(IV/III)	715, 810 [6850]	0.8 ps ^{3}LMCT excited state life time

ND = No data available; [ε] = Extinction coefficient

S12 References

S1. H. J. Shine, B.-J. Zhao, J. N. Marx, T. Ould-Ely, K. H. Whitmire, *J. Org. Chem*. **2004**, *69*, 26, 9255–9261.

S2. K. S. Kjær, N. Kaul, O. Prakash, P. Chábera, N. W. Rosemann, A. Honarfar, O. Gordivska, L. A. Fredin, K.-E. Bergquist, L. Häggström, T. Ericsson, L. Lindh, A. Yartsev, S. Styring, P. Huang, J. Uhlig, J. Bendix, D. Strand, V. Sundström, P. Persson, R. Lomoth, K. Wärnmark, *Science* **2019**, *363*, 249–253.

S3. G. M. Sheldrick, *Acta Cryst A*. **2008**, *64*, 112–122.

S4. P.Gutlich, E. Bill, A.X. Trautwein, *Mößbauer Spectroscopy and Transition Metal Chemistry,* Springer-Verlag Berlin Heidelberg **2011**.

S5. R. Ingalls, *Phys. Rev*. **1964**, *133*, 3A, 787–795.

S6. P. Chábera, Y. Liu, O. Prakash, E. Thyrhaug, A. El Nahhas, A. Honarfar, S. Essén, L. A.Fredin, T. C. B. Harlang , K. S. Kjær, K. Handrup, F. Ericson, H. Tatsuno, K. Morgan, J. Schnadt, L. Häggström, T. Ericsson, A. Sobkowiak, S. Lidin, P. Huang, S. Styring, J. Uhlig, J. Bendix, R. Lomoth, V. Sundström, P. Persson, K. Wärnmark, *Nature* **2017**, *543*, 695−699.

S7. R. T. Azuah, *J. Res. Natl. Inst. Stand. Technol.* **2009**, *114*, 341–358.

S8. K. R. Dunbar, E. J. Schelter, A. V. Palii, S. M. Ostrovsky, V. Y. Mirovitskii, J. M. Hudson, M A. Omary, S. I. Klokishner, B.S. Tsukerblat. *J. Phys. Chem. A* **2003**, *107*, 11102–11111.

S9. M. Zhou, G. P. Robertson, J. Roovers, *Inorg. Chem*. **2005**, *44*, 8317–8325.

S10.M. Reiher, O. Salomon, B. A. Hess, *Theor. Chem. Accounts: Theory Comput. Model. Theor. Chim. Acta* **2001**, *107*, 48-55.

S11.A. D. Becke, *J. Chem. Phys*. **1993**, *98*, 5648–5652.

S12.T. H. Dunning, P. J. Hay, *Gaussian Basis Sets for Molecular Calculations*, Springer, US, Boston, MA **1977**, 1-27.

S13.J. Tomasi, B. Mennucci, R. Cammi, *Chem. Rev*. **2005**, *105*, 2999-3094.

S14.M. Frisch et al., *Gaussian 09, revision C.01*, *Gaussian, Inc., Wallingford CT*, **2016**.

S15.S. Meyer, I. Klawitter, S. Demeshko, E. Bill, F. Meyer, *Angew. Chem. Int. Ed*. **2013**, *52*, 901–905.

S16.I. Nieto, F. Ding, R. P. Bontchev, H. Wang, J. M. Smith, *J. Am. Chem. Soc*. **2008**, *130*, 2716–2717.

S17.M. R. Anneser, G. R. Elpitiya, J. Townsend, E. J. Johnson, X. B. Powers, J. F. DeJesus, K. D. Vogiatzis, D. M. Jenkins, *Angew. Chem. Int. Ed*. **2019**, *58*, 8115–8118.

S18.a) J. J. Scepaniak, J.A. Young, R. P. Bontchev, J. M. Smith, *Angew. Chem. Int. Ed*. **2009**, *48*, 3158–3160; b) J. J. Scepaniak, M. D. Fulton, R. P. Bontchev, E. N. Duesler, M. L. Kirk, J. M. Smith, *J. Am. Chem. Soc*. **2008**, *130*, 10515–10517.

S19.C. Vogel, F. W. Heinemann, J. Sutter, C. Anthon, K. Meyer, *Angew. Chem. Int. Ed*. **2008**, *47*, 2681–2684.

S20.S. A. Cramer, R. H. Sncáhez, D. F. Brakhagea, D. M. Jenkins, *Chem. Commun*., **2014**, *50*, 13967–13970.

S21.M. Malischewski, K. Seppelt, J. Sutter, D. Munz, K. Meyer, *Angew. Chem. Int. Ed*. **2018**, *57*, 14597–14601.

S22.a) B. K. Bower, H. G. Tennent, *J. Am. Chem. Soc*. **1972**, *94*, 2512−2514; b) R. A. Lewis, D. E. Smiles, J. M. Darmon, S. C. E. Stieber, G. Wu, T. W. Hayton, *Inorg. Chem*. **2013**, *52*, 8218−8227.
S23.A. Casitas, J A. Rees, R. Goddard, E. Bill, S. DeBeer, A. Fürstner, *Angew. Chem. Int. Ed*. **2017**, *56*, 10108−10113.
S24.M. Malischewski, M. Adelhardt, J. Sutter, K. Meyer, K. Seppelt, *Science* **2016**, *353*, 678−682.
